# Targeting the mTOR Signaling Pathway in Neuroendocrine Tumors

**DOI:** 10.1007/s11864-014-0294-4

**Published:** 2014-08-05

**Authors:** Jennifer Chan, Matthew Kulke

**Affiliations:** Department of Medical Oncology, Dana-Farber Cancer Institute, 450 Brookline Avenue, Boston, MA 02215 USA

**Keywords:** Neuroendocrine tumor, Carcinoid, Pancreatic neuroendocrine tumor, mTOR inhibitor, Everolimus

## Abstract

Neuroendocrine tumors (NETs) are a heterogeneous group of malignancies characterized by variable but most often indolent biologic behavior. Well-differentiated NETs can be broadly classified as either carcinoid or pancreatic NET. Although they have similar characteristics on routine histologic evaluation, the 2 tumor subtypes have different biology and respond differently to treatment, with most therapeutic agents demonstrating higher response rates in pancreatic NETs compared with carcinoid. Until recently, systemic treatment options for patients with advanced NETs were limited. However, improvements in our understanding of signaling pathways involved in the pathogenesis, growth, and spread of NETs have translated into an expansion of treatment options. Aberrant signaling through the mechanistic pathway of rapamycin (mTOR) pathway has been implicated in neuroendocrine tumorigenesis. Additionally, altered expression of mTOR pathway components has been observed in NETs and has been associated with clinical outcomes. Targeting the mTOR pathway has emerged as an effective treatment strategy in the management of advanced NETs. In a randomized, placebo-controlled study of patients with advanced pancreatic NET, treatment with the mTOR inhibitor everolimus was associated with improved progression-free survival (PFS). Largely based upon these data, everolimus has been approved in the United States and Europe for the treatment of patients with advanced pancreatic NET. The activity of everolimus remains under investigation in patients with carcinoid tumors. In a randomized study of patients with advanced carcinoid tumors associated with carcinoid syndrome, the addition of everolimus to octreotide was associated with improved PFS compared with octreotide. However, the results did not meet the prespecified level of statistical significance based on central review of radiographic imaging. Results from a randomized study examining the efficacy of everolimus in patients with nonfunctional gastrointestinal and lung NETs are awaited. In addition, further investigation is needed to determine whether primary tumor site or other clinical and molecular factors can impact response to mTOR inhibition. Although everolimus can slow tumor progression, significant tumor reduction is rarely obtained. Targeting multiple signaling pathways is a treatment strategy that may provide better tumor control and overcome resistance mechanisms involved with targeting a single pathway. Results of ongoing and future studies will provide important information regarding the added benefit of combining mTOR inhibitors with other targeted agents, such as VEGF pathway inhibitors, and cytotoxic chemotherapy in the treatment of advanced NETs.

## Introduction

Neuroendocrine tumors (NET) are a rare and heterogeneous group of neoplasms that arise from neuroendocrine cells located throughout the body. These tumors are characterized by their ability to secrete peptides resulting in distinctive hormonal syndromes. NETs consist of a spectrum of disease ranging from well-differentiated, low-grade tumors to poorly differentiated, high-grade carcinomas [1•, 2]. In general, poorly differentiated, high-grade carcinomas represent aggressive cancers that have a different natural history and response to treatment compared with well-differentiated, low-grade NETs.

A number of different complex classification systems exist for grading NET pathology [1•]. In the 2010 World Health Organization (WHO) classification, neuroendocrine neoplasms of the digestive system are categorized as low-grade (G1), intermediate-grade (G2), and high-grade (G3) based upon mitotic count and proliferative index (Ki-67) [3]. High grade carcinomas are those with a mitotic count of >20 per 10 high powered fields (HPF) or a Ki-67 proliferation index of >20 %. High grade carcinomas have a more aggressive biology and are generally treated with platinum-based chemotherapy regimens used to treat small cell lung cancer. In contrast, well-differentiated, low- and intermediate-grade NETs have a more indolent biology and lower measures of cell proliferation.

Well-differentiated NETs can be broadly subclassified as either carcinoid or pancreatic NETs. Carcinoid tumors may arise from multiple different organs and historically have been classified according to site of embryonic origin, namely foregut (gastric, bronchial), midgut (small intestine, appendix, proximal large bowel), and hindgut (distal colon, rectum, genitourinary) [4]. While carcinoid and pancreatic NETs may have similar histologic characteristics, these 2 tumor subtypes have different biology and respond differently to therapy, with most agents demonstrating higher response rates in pancreatic NET patients compared with carcinoid.

When NETs are diagnosed at an early stage, surgical resection is often curative. Unfortunately, curative surgery is rarely an option for patients with metastatic disease. Recent studies have demonstrated that in addition to improving symptoms related to hormone hypersecretion, somatostatin analogs slow disease progression in patients with small bowel carcinoid tumors and gastrointestinal neuroendocrine tumors, including pancreatic NET [5, 6]. Treatment approaches with targeted therapy, including the use of agents inhibiting the vascular endothelial growth factor (VEGF) and mTOR signaling pathways and other pathways involved in neuroendocrine tumorigenesis, also provide new therapeutic options for patients with NET [7, 8••].

Notably, there are a subset of patients with NETs that appear histologically well- or moderately differentiated but are associated with Ki-67 proliferation indices >20 % that fall into the high-grade range. The most appropriate therapy for this subgroup of patients has not been well established. A recent series of patients with high-grade gastrointestinal neuroendocrine carcinomas demonstrated that response rates to platinum-based chemotherapy were lower in patients with a Ki-67 <55 % [9]. Because sensitivity to platinum-based chemotherapy appears to be associated with higher Ki-67 proliferation rates, other cytotoxic agents, such as temozolomide, or targeted agents, such as mTOR inhibitors or angiogenesis inhibitors, may play a role in the management of the management of these patients.

The aim of this review is to provide an overview of the role of the mTOR pathway in the pathogenesis of neuroendocrine tumors and to review the role of mTOR inhibitors in the treatment of this disease.

## The mTOR pathway

The mechanistic target (originally referred to as “mammalian target”) of rapamycin (mTOR) is an intracellular serine/threonine kinase that regulates key cell functions involved in cell survival, proliferation, and metabolism. mTOR interacts with several proteins to form 2 multiprotein complexes referred to as mTOR complex 1 (mTORC1) and 2 (mTORC2) [10]. By integrating signals from growth factors and nutrients, mTOR regulates various anabolic and catabolic cellular processes [11, 12•, 13].

**Fig. 1:**
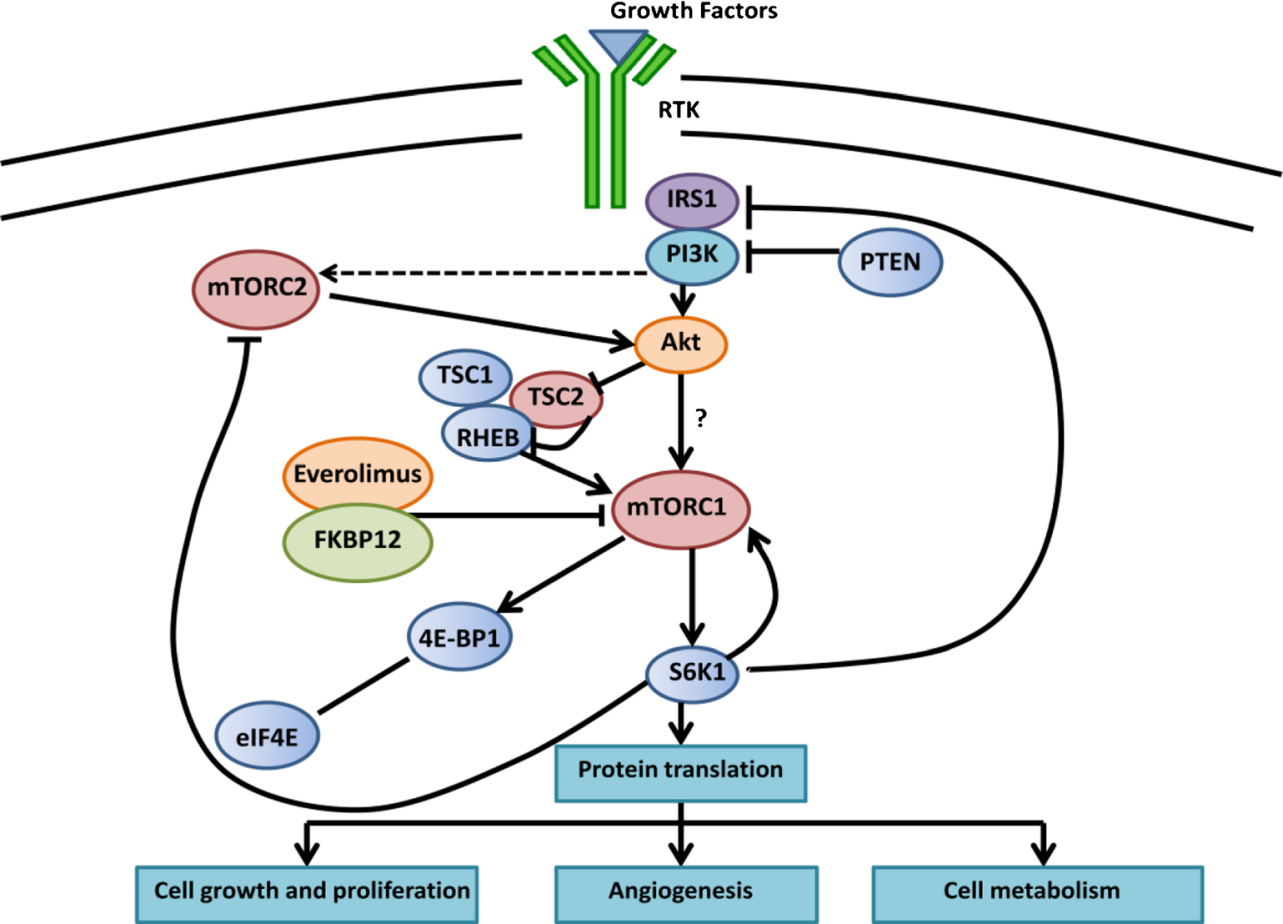
The mTOR signaling pathway. Simplified representation of key components of the mTOR signaling network. The mTOR pathway plays an important role in mediating growth factor signals that stimulate cell growth and proliferation and regulate angiogenesis and cell metabolism. Arrows represent activation; bars represent inhibition. Adapted from Yao et al, 2013 [66].

mTORC1, which is the better characterized of the 2 complexes, is activated by extracellular growth factors and nutrients (Fig. 1). When active, mTORC1 phosphorylates the translational regulators eukaryotic initiation factor 4E (eIF4E) binding protein 1 (4E-BP1) and S6 kinase 1 (S6K1). These events lead to cell proliferation by promoting translation of specific mRNAs encoding proteins regulating cell-cycle progression, angiogenesis, energy metabolism, and metastasis [14]. mTORC1 also promotes lipid biosynthesis and suppresses autophagy through phosphorylation of other key cellular effectors [12•].

Compared with mTORC1, less is known about mTORC2. It also responds to growth factor signals, and when active, mTORC2 regulates cell survival, cytoskeletal remodeling, and cell migration [15, 16]. It also serves to regulate the PI3K/AKT pathway via phosphorylation and activation of Akt [17]. Whereas mTORC1 is sensitive to inhibition by rapamycin, mTORC2 is considered insensitive to rapamycin [12•].

In addition to regulation by energy and nutrient status, the mTOR pathway responds to growth factors through signaling involving the phosphatidylinositol 3-kinase (PI3K) pathway (Fig. 1) [13]. Binding of insulin or insulin-like growth factors to their receptors leads to phosphorylation of insulin receptor substrate (IRS). PI3K is subsequently recruited to the cell membrane, leading to phosphorylation of phosphatidylinositiol-4,5-bisphospate (PIP2) to phosphatidylinositiol-3,4,5-bisphospate (PIP3), and ultimately activation of Akt. The phosphatase PTEN is an inhibitory regulator of the PI3K-Akt-mTOR pathway that antagonizes the action of PI3K by dephosphorylating PIP3 to PIP2, causing suppression of PI3K-dependent cell signaling.

mTOR is linked to the PI3K-Akt pathway by the tuberous sclerosis proteins TSC1 and TSC2, which act as a heterodimer that negatively regulates mTOR signaling. In response to insulin and other growth factors, TSC2 is phosphorylated and inactivated by Akt, which then leads to mTOR activation [18–20].

## The mTOR pathway and pathogenesis of NET

Several observations support the importance of the mTOR pathway in the pathogenesis of NET. First, although most NETs arise sporadically, NETs can arise within the context of several familial cancer syndromes that are due to mutations in genes encoding proteins that lie upstream from mTOR. Neurofibromatosis type 1 (NF-1) and tuberous sclerosis (TS) are autosomal dominant tumor susceptibility syndromes caused by inactivating mutations in the tumor suppressor genes *NF1* and *TSC1* and *TSC2*, respectively [21]. *NF1* encodes the protein neurofibromin, which regulates *TSC1* and *TSC2* [22]. Loss of NF1 in neurofibromatosis leads to constitutive activation of mTOR and is associated with NETs involving the ampulla of Vater, duodenum, and mediastinum. Loss of function of TSC1 and TSC2 leads to mTOR activation in patients with tuberous sclerosis, which has been associated with pancreatic NETs [23].

Second, whole exome sequencing analysis of sporadic pancreatic NETs has identified somatic mutations in genes involved in the mTOR pathway, including PTEN, TS2, and PIK3CA, in 15 % of cases [24•]. Additionally, chromosomal changes, including loss of 16p, the region containing TSC2, and loss of 10q, which contains PTEN, have been reported in pancreatic NET [25, 26].

Altered expression of mTOR pathway components also has been observed in NETs and has been associated with clinical outcomes in several studies. In an analysis of gene expression profiles of 72 primary pancreatic NETs, TSC2 and PTEN were found to be downregulated in most of the primary tumors [27•]. In this study, 85 % of primary tumors showed altered protein levels of TSC2, PTEN, or both. Low levels of expression of TSC2 and PTEN were associated with shorter disease-free and overall survival. Moreover, 8/25 (32 %) patients with low levels of TSC2 and PTEN developed liver metastases and progression of disease compared with none of 20 patients with normal levels of both TSC2 and PTEN. Studies have also demonstrated that expression of mTOR and its downstream targets are associated with clinical outcome [28, 29•]. In an analysis of tumor from 195 patients with NETs arising in various sites, primarily small intestine, expression of mTOR or its activated downstream target *p*-EIF4EBP1 was associated with a higher proliferative index. Furthermore, high expression of mTOR or its activated downstream products were associated with shorter survival [29•].

Interestingly, there appears to be differential expression of mTOR depending on the primary tumor site. Expression levels of mTOR and activation of its downstream targets have been found to be higher in foregut tumors compared with midgut tumors [28]. Additionally, although low expression of PTEN, TSC1, and TSC2 have been found in pancreatic NETs, TSC1 and TSC2 expression appear preserved in small intestinal NET [29•]. This suggests that there may be potential differences in the mechanisms of mTOR activation in different subgroups of NETs.

## Treatment

### Targeting the mTOR pathway

- The mTOR inhibitor rapamycin and its analogs bind FK506 binding protein, and this complex binds to mTORC1, inhibiting downstream signaling [30]. Everolimus and temsirolimus are rapamycin derivatives that have been evaluated in the treatment NET (Tables 1 and 2).

**Table 1:** Clinical trials of mTOR inhibitors in carcinoid tumors

	Agent	No. patients	Tumor response rate (%)	Median TTP or PFS	Reference
Phase II studies					
	Everolimus ^a^	30	17	63 wk	Yao et al. 2008 [37]
	Temsirolimus ^a^	21	5	6.0 mo	Duran et al. 2006 [41]
Phase III studies					
RADIANT-2	Everolimus + octreotide LAR vs.	216	2	16.4 mo	Pavel et al. 2011[38••]
	Placebo + octreotide LAR	214	2	11.3 mo	
RADIANT-4	Everolimus vs.	Ongoing			
	Placebo				

*PFS* progression-free survival, *TTP* time to progression

^a^ Data from the subset of patients with carcinoid tumors in these phase II studies of unselected patients with NET are presented

**Table 2:** Clinical trials of mTOR inhibitors in Pancreatic NET tumors

	Agent	No. patients	Tumor response rate (%)	Median TTP or PFS	Reference
Phase II studies					
	Everolimus^a^	30	27	50 wk	Yao et al. 2008 [37]
RADIANT-1	Everolimus	115	9	9.7 mo	Yao et al. 2010 [31]
	Everolimus + octreotide	45	4	16.7 mo	
	Temsirolimus^a^	15	7	10.6 mo	Duran et al. 2006 [41]
Phase III studies					
RADIANT-3	Everolimus vs.	207	5	11 mo	Yao et al. 2011[8••]
	Placebo	203	2	4.6 mo	
CALGB 80701	Everolimus + octreotide vs.	Ongoing			
	Everolimus + bevacizumab + octreotide				

*PFS* progression-free survival, *TTP* time to progression

^a^ Data from the subset of patients with pancreatic NET in this phase II study of unselected patients with NET are presented

## Everolimus

### Pancreatic NET

- The activity of everolimus in pancreatic NET was explored in the RADIANT-1 trial, an international multicenter phase II trial of 160 patients, 45 of whom also received concurrent treatment with octreotide at the discretion of investigators [31]. Upstream regulation of the IGF pathway is thought to be a potential resistance mechanism for everolimus [32, 33]. Because octreotide has been shown to reduce serum IGF-1 levels in patients with advanced solid tumors, the use of everolimus plus a somatostatin analog to target both upstream and downstream components of the mTOR pathway has been postulated to potentially have greater efficacy than single agent therapy. Among patients receiving octreotide plus everolimus, median PFS was longer compared with those receiving everolimus alone (17 vs 9.7 months). However, whether the addition octreotide to everolimus contributed to higher PFS is uncertain since the study was not randomized or designed to make this comparison.
- Everolimus monotherapy subsequently was compared with best supportive care alone in the placebo-controlled RADIANT-3 trial, which included 410 patients with advanced pancreatic NET [8••]. Approximately 40 % of patients also received somatostatin analog therapy. Everolimus was associated with a significant prolongation in median PFS (11.0 vs 4.6 months, hazard ratio [HR] for progression 0.35, 95 % confidence interval [CI] 0.27–0.45). Confirmed objective partial radiographic responses were observed in 5 % of patients receiving everolimus compared with 2 % of those receiving placebo. The rate of tumor stabilization was high, 73 % among patients receiving everolimus vs 51 % in the placebo group.
- Drug-related adverse among patients with pancreatic NET receiving everolimus included stomatitis, rash, diarrhea, and fatigue [8••]. The most common grade 3 or 4 drug-related adverse events were stomatitis (7 %), anemia (6 %), and hyperglycemia (5 %). Though rare, everolimus has been associated with serious, adverse events, including pneumonitis.
- Everolimus causes hyperglycemia, particularly in those with pre-existing hyperglycemia. In the RADIANT-3 trial, the frequency of severe (grade 3 or 4) hyperglycemia was higher in those with pre-existing diabetes mellitus or baseline hyperglycemia (15 % vs 3 % in those without diabetes or baseline hyperglycemia) [34]. Because of this effect, everolimus may be of particular value in patients with hypoglycemia related to insulinoma [35, 36]. In 1 report, 4 patients with malignant insulinoma and refractory hypoglycemia experienced normalization of glucose levels while receiving everolimus [35]. Two of these patients had an objective radiographic antitumor response, which may have led to improvements in insulin secretion. Clinical improvement in the 2 patients with stable disease suggests a possible direct effect of everolimus on insulin production and/or release or an effect on peripheral insulin sensitivity.

### Carcinoid tumors

- Everolimus has been evaluated in combination with octreotide in a phase II study of patients with advanced NETs. Partial responses were observed in 5 of 30 (17 %) patients with carcinoid tumors, with a median PFS of 63 weeks in this group of patients [37].
- The activity of everolimus was further investigated in patients with functional NET in the randomized, placebo-controlled RADIANT-2 trial [38••]. In this study, 429 patients with advanced NETs associated with carcinoid syndrome and radiographic disease progression in the preceding 12 months were randomly assigned to octreotide LAR with either everolimus or placebo. Half of patients had a primary small bowel tumor; lung primary tumors were the second most common tumor type. The median PFS as assessed by central radiology review was 16.4 months for patients receiving everolimus and octreotide LAR compared with 11.3 months for patients receiving placebo and octreotide LAR (HR 0.77, 95 % CI 0.59–1.00; *P* = 0.026). These results did not meet the prespecified level of statistical significance. However, based on local investigator radiology assessment, combined therapy was associated with a median PFS duration of 12.0 months compared with 8.6 months with placebo (HR 0.78, 95 % CI 0.62–0.98; *P* = 0.018). Additionally, imbalances in prognostic variables favoring the control group, including disease site and performance status, could have affected the primary outcome results. A subsequent analysis found a significant PFS benefit for everolimus after adjusting for randomization imbalances (HR for progression 0.62, 95 % CI 0.51–0.87, *P* = 0.003) [39].
- The best overall radiographic response was a partial response in 2 % of both groups; stable disease was the best response in 84 % of the patients treated with everolimus and 81 % of patients receiving placebo [38••]. Patients treated with everolimus had a higher rate of biochemical responses. Serum chromogranin A levels decreased in 46 % of patients receiving everolimus compared with 36 % of patients receiving placebo, and 24 hour urinary 5-hydroxyindoleacetic acid (5-HIAA) excretion decreased in 61 % of patients receiving everolimus compared with 54 % of patients receiving placebo. Data on control of symptoms related to carcinoid syndrome were not reported.
- Additional studies are also needed to determine whether primary tumor site of origin may impact response to everolimus. Patients with advanced colorectal NET have a particularly poor prognosis. In a post-hoc analysis of the RADIANT-2 study, patients with colorectal NETs receiving everolimus plus octreotide LAR had significantly longer PFS (29.9 mo; *n* = 19) compared with those receiving placebo plus octreotide LAR (6.6 mo; *n* = 20) [40]. Furthermore, some degree of tumor shrinkage was more frequently noted in patients receiving everolimus plus octreotide LAR compared with those receiving placebo plus octreotide LAR (67 % vs 37 %).
- The RADIANT-4 trial, a phase III study in which patients with advanced, nonfunctional lung or gastrointestinal NETs were randomized to receive everolimus or placebo, recently completed accrual (clinical trials.gov, NCT01524783). The results of this study will provide important information regarding the activity of everolimus in the treatment of nonpancreatic NET.

## Temsirolimus

- The single-agent activity of temsirolimus was evaluated in a multicenter phase II study of 37 patients with advanced, progressive neuroendocrine tumors [41]. Although the intent-to-treat response rate for the cohort was low (6 %), 54 % of patients experienced stable disease while on treatment with a median time to progression (TTP) of 6 months. Higher baseline tumor levels of phosphorylated mTOR predicted for better outcomes. Furthermore, temsirolimus appeared more active in patients with pancreatic NET compared with carcinoid; median TTP in patients with pancreatic NET was 10.6 months compared with 6 months in the carcinoid subgroup. However, the small size of this study limits definite conclusions regarding the impact of primary tumor site on efficacy of temsirolimus.

## Future directions with mTOR inhibitor therapy

- Rapamycin and its derivatives are generally cytostatic rather than cytotoxic. One of the factors contributing to their limited clinical success is the existence of multiple feedback loops regulating cell survival (Fig. 1). Under normal circumstances, mTORC-1 activation of S6K1 promotes degradation of insulin receptor substrate (IRS), leading to attenuation of PI3K signaling. Inhibition of mTORC1 can lead to increased PI3K signaling by relieving this negative feedback [33, 42]. In addition, mTORC1-mediated signaling can inhibit mTORC2 through phosphorylation of rictor, one of the components of mTORC2. By blocking this feedback loop, rapamycin can contribute to mTORC2-mediated AKT activation. Furthermore, studies have also demonstrated that inhibition of the PI3K/AKT/mTOR pathway can result in activation of other receptor tyrosine kinases, resulting in downstream signaling promoting cell growth [43].
- Targeting multiple signaling pathways may provide better tumor control and overcome resistance mechanisms. Combining an mTOR inhibitor with somatostatin analogs, inhibitors of the VEGF pathway and cytotoxic chemotherapy have been evaluated as treatment strategies for NETs.

## Combining mTOR inhibitor and somatostatin analog

- Because octreotide has been shown to decrease IGF-1 levels and PI3K/Akt signaling in vitro, it has been postulated that combining an mTOR inhibitor with a somatostatin analog might result in enhanced antitumor activity [44]. Everolimus has been evaluated in combination with octreotide in several studies, including patients with pancreatic NET in stratum 2 of the RADIANT-1 trial and patients with carcinoid tumors in the phase III RADIANT-2 trial. In the RADIANT-1 trial, patients receiving octreotide and everolimus had longer PFS compared with patients receiving everolimus monotherapy [31]. However, the study was not randomized or designed to make this comparison. In the RADIANT-2 trial, although combined therapy with everolimus and octreotide was associated with a significantly longer PFS duration compared with everolimus and placebo based on local investigator radiology review, the improvement in PFS was not statistically significant according to central radiology review [38••]. Further investigation is needed to determine whether there are specific subsets of patients with advanced NETs who benefit most from the addition of everolimus to octreotide.
- Pasireotide is a novel somatostatin analog that binds to a broader range of somatostatin receptor subtypes (sst) than octreotide. Compared with octreotide, pasireotide has a greater binding affinity to sst1, sst3, and sst5 and comparable affinity with sst2 [45]. Increased receptor binding may lead to additional antiproliferative activity and growth inhibition in NET [44]. A phase I study has established the feasibility of combining pasireotide and everolimus [46]. Hyperglycemia was a commonly observed toxicity. A partial radiographic tumor response was noted in 1/21 patients (5 %), and 17/21 (81 %) experienced at least some tumor regression as the best response to therapy. The COOPERATE-2 study, a multi-center randomized phase II study, recently completed accrual and has examined the combination of everolimus alone or in combination with pasireotide LAR in patients with advanced, progressive pancreatic NET (clinical trials.gov, NCT01374451). The results of this study will provide additional information regarding the added efficacy of combining an mTOR inhibitor with a somatostatin analog.

## Combining mTOR inhibitor and VEGF pathway inhibitor

- A key role for angiogenesis and VEGF pathway signaling in NET is suggested by clinical observations that neuroendocrine tumors are vascular tumors. Expression of VEGF has been demonstrated in carcinoid and pancreatic NETs [47, 48]. Increased expression of VEGF receptor-2 (VEGFR-2) has been demonstrated in tissue from gastrointestinal carcinoid tumors and a carcinoid cell line [49, 50]. Additionally, pancreatic neuroendocrine tumors also show widespread expression of VEGFR-2 and -3 in addition to platelet-derived growth factor receptors (PDGFRs) α and β, stem-cell factor receptor (c-kit) [51–53].
- The tyrosine kinase inhibitor sunitinib has shown activity against a range of signaling pathways and growth factors/receptors, including VEGFR-1, -2 and -3, PDGFR-α and -β, KIT, RET, FMS-like tyrosine kinase-3 (FLT3), and colony-stimulating factor receptor (CSF-1R). In a randomized phase III study examining the activity of sunitinib in patients with progressive pancreatic NET, sunitinib was associated with a median progression-free survival (PFS) of 11.4 months, as compared with 5.5 months for placebo (*P* < .001) [7]. Two other small molecule tyrosine kinase inhibitors (TKIs), sorafenib, and pazopanib, have also been evaluated in phase II studies [54, 55]. Although response rates to TKIs in carcinoid tumors have been low, all studies report a high rate of disease stabilization and potentially encouraging progression-free survival durations.
- Bevacizumab, a monoclonal antibody against VEGF, has been evaluated in a randomized phase II study of patients with advanced or metastatic carcinoid tumors on a stable dose of octreotide. Patients were randomly assigned to receive 18 weeks of bevacizumab or pegylated IFN-α 2b [56]. During the first 18 weeks of therapy, 18 % of the bevacizumab-treated patients experienced radiographic partial responses, and 77 % had stable disease. Furthermore, after 18 weeks, 95 % of patients treated with octreotide plus bevacizumab remained progression-free compared with only 68 % of those receiving octreotide plus IFN-α.
- A number of recently completed and ongoing studies have evaluated the combination of an mTOR inhibitor with inhibitors of the VEGF pathway. Combining everolimus with tyrosine kinase inhibitors of VEGFR and other growth factor receptors may be limited by toxicity. In a phase I study of everolimus in combination with sorafenib, dose-limiting toxicity precluding escalation to full doses of each agent was observed [57]. However, the combination of the everolimus and bevacizumab was shown to be well tolerated and associated with antitumor activity (overall response rate 26 %) in an initial phase II study enrolling patients with low or intermediate grade neuroendocrine tumors [58]. Furthermore, encouraging early results have been noted in a phase II trial of temsirolimus plus bevacizumab in 55 patients with progressive NET. In a preliminary report, a confirmed partial response was documented in 20 patients (37 %), and 44 (80 %) remained progression-free at 6 months [59]. Results of CALGB 80701, a phase II trial of patients with advanced pancreatic NETs randomized to receive either everolimus and octreotide or everolimus plus bevacizumab and octreotide, will provide additional information about the benefits of combined mTOR and VEGF pathway inhibition (clinical trials.gov, NCT01229943).

## Combining mTOR inhibitor and cytotoxic chemotherapy

- Cytotoxic chemotherapy has minimal activity in patients with advanced carcinoid tumors. In contrast, pancreatic NETs may respond well to treatment with streptozocin and other alkylating agents [60, 61]. Recent prospective and retrospective studies have suggested that temozolomide-based regimens may be comparable in efficacy and more tolerable than streptozocin-based regimens. In retrospective series, temozolomide-based therapy has been associated with overall response rates of 8 %–70 % in patients with advanced pancreatic NET [62–64].
- The combination of temozolomide and everolimus has been evaluated in a phase I/II study of patients with advanced pancreatic NET [65]. Treatment was associated with known side effects of each drug without evidence of synergistic toxicity. Encouraging evidence of antitumor activity with this combination was observed. Among 40 evaluable patients, 16 (40 %) experienced a partial response. The median PFS duration was 15.4 months, which is superior to the reported PFS observed with everolimus alone in the randomized, placebo-controlled RADIANT-3 study. However, these results need to be interpreted with caution since this was a single-arm study. Furthermore, disease progression prior to study enrollment was not a requirement in this study, as it was in the RADIANT-3 study. Future studies evaluating the relative efficacy of combining chemotherapy with an mTOR inhibitor compared with treatment with either agent alone are warranted.

## Conclusions

Recent improvements in our understanding of the molecular biology of NETs have led to an expansion of treatment options for patients with advanced disease. Studies indicate that the mTOR pathway plays an important role in the pathogenesis of NET. The mTOR inhibitor everolimus has been shown to significantly delay disease progression in patients with pancreatic NET. Further studies evaluating everolimus in advanced carcinoid are anticipated. Results of ongoing and future studies will provide important information regarding the added benefit of combining an mTOR inhibitor with cytotoxic chemotherapy and other targeted agents, such as VEGF pathway inhibitors, in the treatment of advanced NETs.
